# The Activity of SN33638, an Inhibitor of AKR1C3, on Testosterone and 17β-Estradiol Production and Function in Castration-Resistant Prostate Cancer and ER-Positive Breast Cancer

**DOI:** 10.3389/fonc.2014.00159

**Published:** 2014-06-18

**Authors:** Yarong Diana Yin, Melissa Fu, Darby G. Brooke, Daniel M. Heinrich, William A. Denny, Stephen M. F. Jamieson

**Affiliations:** ^1^Auckland Cancer Society Research Centre, The University of Auckland, Auckland, New Zealand; ^2^Maurice Wilkins Centre for Molecular Biodiscovery, The University of Auckland, Auckland, New Zealand

**Keywords:** AKR1C3, SN33638, castration-resistant prostate cancer, ER-positive breast cancer, 11β-prostaglandin F_2α_, testosterone, 17β-estradiol, prostate-specific antigen

## Abstract

AKR1C3 is a novel therapeutic target in castration-resistant prostate cancer (CRPC) and estrogen receptor (ER)-positive breast cancer because of its ability to produce testosterone and 17β-estradiol intratumorally, thus promoting nuclear receptor signaling and tumor progression. A panel of CRPC, ER-positive breast cancer and high/low AKR1C3-expressing cell lines were treated with SN33638, a selective inhibitor of AKR1C3, in the presence of hormone or prostaglandin (PG) precursors, prior to evaluation of cell proliferation and levels of 11β-PG F_2α_ (11β-PGF_2α_), testosterone, 17β-estradiol, and prostate-specific antigen (PSA). A meta-analysis of *AKR1C3* mRNA expression in patient samples was also conducted, which revealed that *AKR1C3* mRNA was upregulated in CRPC, but downregulated in ER-positive breast cancer. 11β-PGF_2α_ and testosterone levels in the cell line panel correlated with AKR1C3 protein expression. SN33638 prevented 11β-PGF_2α_ formation in cell lines that expressed AKR1C3, but partially inhibited testosterone formation and subsequently cell proliferation and/or PSA expression only in high (LAPC4 AKR1C3-overexpressing cells) or moderate (22RV1) AKR1C3-expressing cell lines. SN33638 had little effect on 17β-estradiol production or estrone-stimulated cell proliferation in ER-positive breast cancer cell lines. Although SN33638 could prevent 11β-PGF_2α_ formation, its ability to prevent testosterone and 17β-estradiol production and their roles in CRPC and ER-positive breast cancer progression was limited due to AKR1C3-independent steroid hormone production, except in LAPC4 AKR1C3 cells where the majority of testosterone was AKR1C3-dependent. These results suggest that inhibition of AKR1C3 is unlikely to produce therapeutic benefit in CRPC and ER-positive breast cancer patients, except possibly in the small subpopulation of CRPC patients with tumors that have upregulated *AKR1C3* expression and are dependent on AKR1C3 to produce the testosterone required for their growth.

## Introduction

Hormone-dependent malignancies, such as breast and prostate cancer, generally require intracellular concentrations of steroid hormones for nuclear receptor activation to trigger downstream signaling and promote tumor growth (1, 2). However, in castration-resistant prostate cancer (CRPC) in men and estrogen receptor (ER)-positive breast cancer in post-menopausal women, the levels of circulating hormones are diminished. Tumors can overcome this by producing intracrine hormones from circulating precursors to activate nuclear receptor signaling (3–5).

The oxidoreductase AKR1C3, also known as 17β-hydroxysteroid dehydrogenase (17β-HSD) type 5, has been implicated in the development of CRPC (4, 6–9), and ER-positive breast cancer (10, 11), because it can produce androgens locally in these tissues. AKR1C3 catalyzes the production of potent androgens testosterone and 5α-dihydrotestosterone in the prostate by reducing the androgen precursors Δ^4^-androstene-3,17-dione (12), 5α-androstane-3,17-dione (13), or androsterone via 3α-diol (14). In the breast, AKR1C3 can also reduce the weak estrogen estrone to the potent estrogen 17β-estradiol (10, 12, 14).

*AKR1C3* mRNA has been reported to be upregulated in metastatic and non-metastatic CRPC compared to local prostate carcinoma (4, 8, 9, 15) and the subsequent *de novo* synthesis of androgens in the prostate can drive androgen receptor (AR) activation and may be responsible for the development of resistance to androgen deprivation therapy in CRPC patients (6, 8, 16). The expression of *AKR1C3* in ER-positive breast cancer is less clear. Although *AKR1C3* has been reported to be upregulated in pre-invasive and malignant breast cancer tissues compared to normal breast tissue (17, 18), and its expression shown to correlate with poor prognosis and an increased rate of late recurrence (18, 19), other studies have found variable or downregulated *AKR1C3* expression in breast cancer tissues (20, 21).

AKR1C3 also functions in a steroid-independent manner as a prostaglandin (PG) F synthase to convert PGH_2_ to PGF_2α_ and PGD_2_ to 9α, 11β-PGF_2α_ (22, 23), an activity that has been shown to prevent the differentiation of human myeloid leukemia cells (24, 25). Furthermore, AKR1C3 has been reported to have roles in xenobiotic metabolism as a carbonyl reductase (26, 27), in the oxidation of polycyclic aromatic hydrocarbons (28, 29), and in the aerobic activation of the hypoxia-activated prodrug PR-104 (30, 31).

Due to the numerous enzymatic activities of AKR1C3, its pattern of activity in tissues is determined by its distribution, its catalytic efficiency for the substrate, the availability of the substrate, and its regulation by steroid hormone levels or the antioxidant response transcription factor Nrf2 (8, 30, 32, 33). The preferred action of AKR1C3 *in vitro*, as determined by its catalytic efficiency, is its PGD_2_ 11-ketoreductase activity (10, 22, 33). However, in tissues with high enzyme and steroid substrate expression, such as the breast and prostate (12, 34), 17β-HSD activity may still be prevalent despite its lower catalytic efficiency relative to PGD_2_ 11-ketoreductase activity (10).

**Figure 1:**
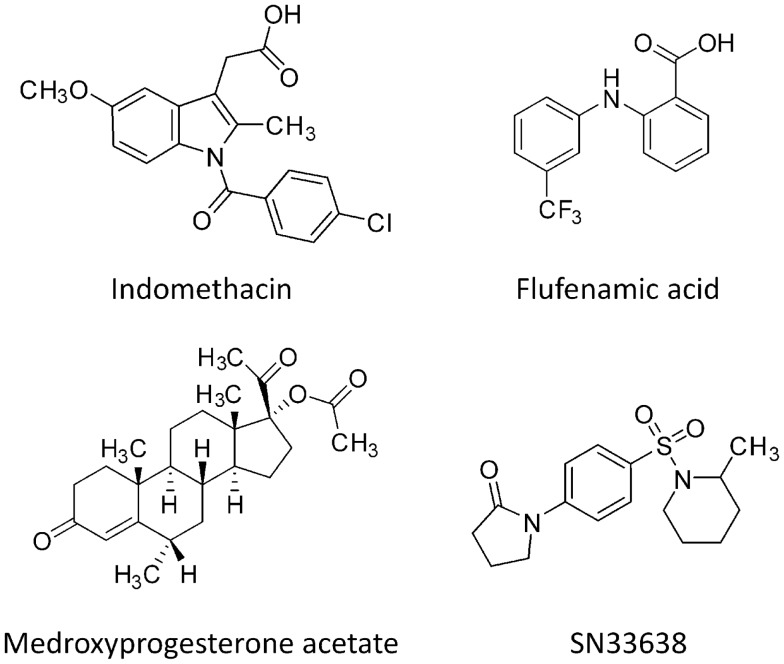
**Chemical structures of AKR1C3 inhibitors**.

We recently reported the synthesis of a novel series of *N*-(4-(2-oxopyrrolidin-1-yl)phenyl)piperidine-1-sulfonamides as potent and isoform-selective inhibitors of AKR1C3 (35). The lead compound, SN33638, had low nanomolar potency against AKR1C3 and >300-fold selectivity for AKR1C3 over the other AKR1C isoforms. This compared favorably with other drugs with AKR1C3 inhibitory activity, such as non-steroidal anti-inflammatory drugs (NSAIDs; e.g., indomethacin and flufenamic acid), which are less potent against AKR1C3 and are non-selective for cyclooxygenase enzymes (36, 37) and medroxyprogesterone acetate (MPA), which is non-selective for the other AKR1C isoforms (38) (Figure 1). Here, we investigate AKR1C3 expression and activity across a panel of CRPC and ER-positive breast cancer cell lines. We use this cell line panel to evaluate the ability of SN33638 to inhibit 11β-PGF_2α_, testosterone, and 17β-estradiol production, and to determine the effect of AKR1C3 inhibition on cell proliferation and prostate-specific antigen (PSA) expression.

## Materials and Methods

### Chemicals

SN33638 was synthesized as described previously (35). Androstenedione, estrone, indomethacin, flufenamic acid, medroxyprogesterone acetate, and apigenin, were purchased from Sigma-Aldrich, 5-(3-bromo-4-hydroxybenzylidene)-3-(4-methoxyphenyl)-2-thioxothiazolidin-4-1 (17β-HSD3 inhibitor) was purchased from Merck Millipore and PGD_2_ was purchased from Cayman Chemical Company.

### Cell culture

The cell lines used are described in Table 1, including their hormone sensitivity and receptor status as previously characterized (39, 40). All cell lines were sourced from ATCC, except LAPC4 and PC3, which were kindly supplied by Dr. William Aronson and Dr. Ronnie Cohen, respectively. HCT116, T47D, and LAPC4 cells were transfected with a plasmid encoding open reading frames for *AKR1C3* that was cloned into an F279-V5^puro^ Gateway^®^-compatible vector as described previously (30, 41) using FuGENE^®^ HD Transfection Reagent (Roche). Cell lines were maintained in αMEM supplemented with 5% FCS (Moregate Biotech) (HCT116, NCI-H460), 10% FCS, and 1% PSG (penicillin–streptomycin–glutamine; Life Technologies) (22RV1, PC3, DU145, LNCaP) or 10% FCS and 0.01–0.02 mg/mL human insulin (Sigma-Aldrich) (MCF7, T47D), RPMI with 10% FCS (HCC1500), IMDM with 10% FCS and 1% PSG (LAPC4), or DMEM with 10% non-heat inactivated FCS (VCaP). Transfected cell lines were further supplemented with 0.5–1.0 μM puromycin (Life Technologies). For drug treatments, cells were seeded in phenol red-free media (αMEM, RPMI, IMDM, or DMEM as above) supplemented with 5% charcoal-stripped serum (Life Technologies). All media, except phenol red-free DMEM (Sigma-Aldrich), were purchased from Life Technologies. LAPC4, LAPC4 AKR1C3, VCaP, and HCC1500 cells were maintained in poly-d-lysine (Becton Dickinson) coated flasks and 96-well plates.

**Table 1:** **Cell line type, hormone-dependence, and receptor status**.

Cell Line	Tumor type	Origin	Hormone-dependence	Receptor status
HCT116	Colorectal	Primary	–	–
NCI-H460	Large cell lung	Pleural effusion	–	–
LAPC4	Prostate	Lymph node^a^	Androgen sensitive	AR-positive
VCaP	Prostate	Vertebrae^a^	Androgen sensitive	AR-positive
22RV1	Prostate	Primary^a^	Androgen sensitive	AR-positive (H874Y mutant)
PC3	Prostate	Vertebrae	Androgen insensitive	AR-negative
DU145	Prostate	Brain	Androgen insensitive	AR-negative
LNCaP	Prostate	Lymph node	Androgen sensitive	AR-positive (T877A mutant)
MCF7	Invasive ductal carcinoma (breast)	Pleural effusion	Estrogen sensitive	ER-positive
T47D	Invasive ductal carcinoma (breast)	Pleural effusion	Estrogen sensitive	ER-positive
HCC1500	Ductal carcinoma (breast)	Primary	Estrogen sensitive	ER-positive

*^a^Cell line established from xenograft*.

### Oncomine analysis

*AKR1C3* and *NQO1* gene expression were analyzed using the publicly accessible online database Oncomine (Compendia Biosciences). mRNA expression was analyzed in all prostate cancer datasets that contained CRPC samples (Tamura Prostate, Tomlins Prostate, Holzbeierlein Prostate, Varambally Prostate, Best Prostate 2, Chandran Prostate, Grasso Prostate), all breast cancer datasets that had normal breast samples, and breast cancer samples with known hormone status (Curtis Breast, TCGA Breast, Richardson Breast 2, Gluck Breast, Ma Breast 4, Turashvili Breast, and Zhao Breast) and in the Barretina cell line dataset. The *AKR1C3* or *NQO1* mRNA expression value for each sample was normalized to the median expressed probeset for that particular sample.

### Western blotting

Cell lysis was carried out for each cell line in modified radioimmunoprecipitation assay lysis buffer containing 1% protease inhibitor cocktail (Sigma-Aldrich) on ice for 30 min. Cells were centrifuged at 13,000 rpm for 5 min at 4°C to remove insoluble material. Protein concentration of cell lysates was determined by bicinchoninic acid assay (Sigma-Aldrich) against bovine serum albumin (BSA; Immuno-Chemical Products Ltd.) standards. Twenty micrograms of each lysate was loaded onto NuPAGE^®^ Novex 4–12% Bis–Tris pre-cast 10-well gels (Life Technologies) and separated by SDS-PAGE at 120 V for 90 min. Each gel was transferred onto a nitrocellulose membrane (Bio-Rad Laboratories) at 100 V for 70 min and incubated in blocking buffer [PBS with 0.5% Tween^®^-20 (Serva) and 5% BSA] for 1 h. Membranes were washed in PBS with 0.5% Tween^®^-20 then cut and incubated overnight at 4°C with monoclonal antibodies against either AKR1C3 (clone NP6.G6.A6; Sigma-Aldrich) at a 1:5000 dilution in 5% BSA or α-tubulin (clone B-5-1-2; Sigma-Aldrich) at a dilution of 1:5000 in 5% BSA. Membranes were washed then incubated with goat anti-mouse IgG HRP-conjugated secondary antibody (Santa Cruz Biotechnology) at 1:10,000 dilutions in blocking buffer for 1 h at room temperature. After further washes, the membranes were incubated with SuperSignal^^®^^ West Pico chemiluminescent substrate (Thermo Scientific) for 5 min prior to imaging on a LAS-3000 luminescent image analyzer (Fujifilm). Western blots were quantitated using ImageJ 1.45s software (NIH).

### Measurement of 11β-PGF_2α_, testosterone, and 17β-estradiol by ELISA

Cells were seeded into 96-well plates in hormone-deprived media at appropriate seeding densities [10,000–50,000 cells per well for all lines except HCC1500 (100,000 cells per well; 11β-PGF_2α_ ELISA)] to reach approximately 70% confluency at the time of sampling and were left to settle for 24 h at 37°C and 5% CO_2_. Inhibitors were added to the plates at single or multiple concentrations for 1 h prior to administration of equimolar (28 nM) concentrations of PGD_2_ (11β-PGF_2α_ ELISA), androstenedione (testosterone ELISA), or estrone (17β-estradiol ELISA). The plates were returned to the incubator for a further 24 h before cell supernatant was removed for analysis of 11β-PGF_2α_, testosterone, or 17β-estradiol by ELISA as per manufacturer’s instructions (11β-PGF_2α_ and testosterone ELISA, Cayman Chemical Company; 17β-estradiol ELISA, Oxford Biomedical Research), and cells were fixed for sulforhodamine B analysis as described below. Each ELISA plate was read at a wavelength of 405 nM (11β-PGF_2α_ and testosterone) or 650 nM (17β-estradiol) on a SpectraMax M2 microplate reader (Molecular Devices) and quantitated against a standard curve generated in hormone-deprived culture media. Cross-reactivity to PGD_2_, androstenedione, or estrone was accounted for by adding these to cell-free wells of each ELISA plate.

### Cell proliferation

Cells were seeded into 96-well plates in hormone-deprived media at appropriate seeding densities (5000–20,000 cells per well) to reach approximately 70% confluency at the time of sampling and left to settle for 48 h at 37°C and 5% CO_2_. SN33638 was added to the plates immediately prior to stimulation with multiple concentrations of androstenedione or estrone. The plates were returned to the incubator for 5 days before fixing in 10% trichloroacetic acid (Merck Millipore) at 4°C for 1 h and staining with 0.4% sulforhodamine B (Sigma-Aldrich) in 1% acetic acid for 30 min in the dark at room temperature. Plates were washed in 1% acetic acid, dried, and incubated with unbuffered Tris base (10 mM; Serva) for 30 min on a plate shaker in the dark to solubilize the stain. The plates were read on a BioTek EL808 microplate reader at an absorbance of 490 nM with a reference wavelength of 450 nM.

### Measurement of PSA concentrations

Cells were seeded at 20,000 cells per well (LAPC4, LAPC4 AKR1C3) or 50,000 cells per well (22RV1) into 96-well plates in hormone-deprived media and left to settle for 24 h at 37°C and 5% CO_2_. SN33638 was added to the plates 1 h prior to multiple concentrations of androstenedione. The plates were returned to the incubator for 24 h, before the cell supernatant was removed and frozen for PSA analysis. Determination of total PSA concentration was carried out at LabPLUS, Auckland City Hospital, by immunoassay on a Cobas^®^ e601 analyzer according to manufacturer’s instructions (Roche).

## Results

### *AKR1C3* mRNA is upregulated in CRPC patients but downregulated in breast cancer patients

**Figure 2:**
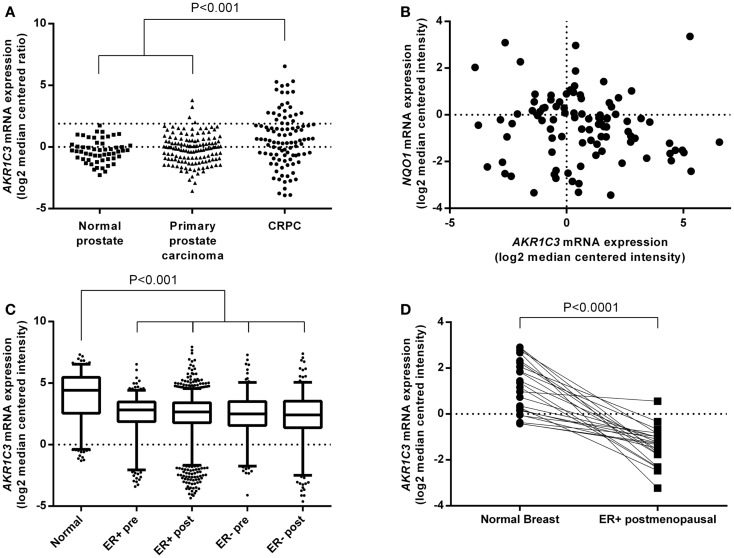
***AKR1C3* mRNA expression is upregulated in tumor samples from CRPC patients but downregulated in breast cancer patient tumors**. **(A)** Meta-analysis of *AKR1C3* mRNA expression in normal prostate (*n* = 54), primary prostate carcinoma (*n* = 133), and CRPC (*n* = 100) tissue in patients from seven Oncomine prostate cancer datasets. The upper dotted line indicates 75th percentile for CRPC. **(B)** Correlation of *NQO1* mRNA expression with *AKR1C3* mRNA expression in CRPC patients from **(A)**. **(C)** Meta-analysis of *AKR1C3* mRNA expression in normal breast (Normal, *n* = 257), premenopausal ER-positive breast cancer (ER+ pre, *n* = 281), post-menopausal ER-positive breast cancer (ER+ post, *n* = 1337), premenopausal ER-negative breast cancer (ER− pre, *n* = 189), and post-menopausal ER-negative breast cancer (ER− post, *n* = 278) patients in seven Oncomine breast cancer datasets. The line in each box represents the median, the lower, and upper boundaries represent the 25th and 75th percentiles, and the whiskers show the 5th and 95th percentiles. **(D)** *AKR1C3* mRNA expression in paired normal and ER-positive breast cancer samples in post-menopausal women (*n* = 23) from **(C)**. Significance of differences was evaluated by one-way ANOVA with Dunnett’s multiple comparison analysis **(A,C)** or by Student’s *t*-test **(D)**.

We analyzed the online microarray database Oncomine to determine the *AKR1C3* mRNA expression levels in CRPC and breast cancer patients relative to normal tissue or primary disease. Meta-analysis of seven prostate cancer datasets that included normal prostate or primary prostate carcinoma samples in addition to CRPC revealed that *AKR1C3* expression is significantly upregulated in CRPC relative to both normal prostate and primary prostate carcinoma (*P* < 0.001; one-way ANOVA with Dunnett’s multiple comparison analysis; Figure 2A). The magnitude of this difference was most evident in the highest *AKR1C3*-expressing samples, where the upper quartile of CRPC corresponded to the 97th percentile of primary prostate carcinoma and above the 100th percentile for normal prostate. Since *AKR1C3* is regulated by Nrf2 (32), we also analyzed the samples for mRNA expression of *NQO1*, a reporter of Nrf2 activity (42, 43), to determine if the upregulation of *AKR1C3* in CRPC samples was driven by increased Nrf2 activity. *NQO1* mRNA levels were unchanged in CRPC relative to normal prostate or primary prostate carcinoma (data not shown) and there was no significant correlation between *NQO1* mRNA and *AKR1C3* mRNA expression in CRPC patients (*R*^2^ = 0.02; Pearson product-moment correlation; Figure 2B). A similar meta-analysis was conducted across seven breast cancer datasets that included normal breast samples, showing that *AKR1C3* mRNA expression is significantly downregulated in breast cancer samples relative to normal breast tissue (*P* < 0.001; one-way ANOVA with Dunnett’s multiple comparison analysis; Figure 2C), including in paired samples from post-menopausal ER-positive breast cancer patients (*P* < 0.0001) (Figure 2D).

### AKR1C3 protein expression is variable in prostate and breast cancer cell lines

**Figure 3:**
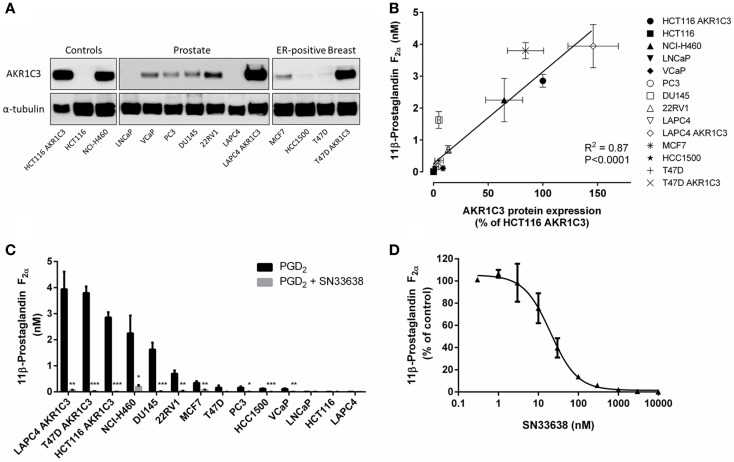
**PGD_2_ 11-ketoreductase activity correlates with AKR1C3 protein expression and can be inhibited by SN33638**. **(A)** AKR1C3 and α-tubulin protein expression by western blotting in the cell line panel. **(B)** Correlation between 11β-PGF_2α_ formation following stimulation with 28 nM PGD_2_ (*n* = 2–6) and AKR1C3 protein expression (average of two to three independent lysates) in the cell line panel. **(C)** 11β-PGF_2α_ formation in the cell line panel after stimulation with PGD_2_ in the presence or absence of 10 μM SN33638 (*n* = 2–6). **(D)** EC_50_ plot of inhibition of 11β-PGF_2α_ production by SN33638 following stimulation with PGD_2_ in HCT116 AKR1C3 cells (*n* = 2–3). Bars or symbols represent the mean ± SEM. Statistical significance of correlation analysis was evaluated by Spearman’s rank-order correlation and of differences between mean values by Student’s *t*-test. *P < 0.05; **P < 0.01; ***P < 0.001 vs. no inhibitor controls.

We generated a cell line panel of prostate and ER-positive breast cancer cell lines and evaluated AKR1C3 protein expression by western blotting in comparison to cell lines with high (NCI-H460 NSCLC and HCT116 AKR1C3-overexpressing colon cancer cells) and low (HCT116 wild-type) AKR1C3 expression. To ensure the panel included CRPC and ER-positive breast cancer cell lines with high AKR1C3 expression, we transfected LAPC4 and T47D cells with an AKR1C3 plasmid to generate AKR1C3-overexpressing cell lines. The stably transfected cell lines had high protein expression of AKR1C3, similar to NCI-H460 and HCT116 AKR1C3, in contrast to the wild-type lines (LAPC4 and T47D), which had very low AKR1C3 expression (Figure 3A). Moderate protein expression was observed in 22RV1, VCaP, PC3, and DU145 prostate cancer cells and in MCF7 breast cancer cells. Overall, the protein expression in the cell line panel correlated well with the mRNA expression data for these cell lines reported in the Barretina cell line dataset in Oncomine (*R* = 0.778; *P* < 0.01; Spearman’s rank-order correlation).

### SN33638 prevents the PGD_2_ 11-ketoreductase activity of AKR1C3

The PGD_2_ 11-ketoreductase activity of AKR1C3 was determined by stimulating the cell line panel with PGD_2_ for 24 h, then detecting 11β-PGF_2α_ levels by ELISA. The formation of 11β-PGF_2α_ in the cell line panel correlated well with AKR1C3 protein expression (*R* = 0.907, *P* < 0.0001; Spearman’s rank-order correlation; Figure 3B). Addition of the AKR1C3 inhibitor SN33638 at 10 μM dramatically reduced 11β-PGF_2α_ levels by ≥80% in all cell lines that expressed AKR1C3 (Figure 3C). Varying concentrations of SN33638, MPA, indomethacin, and flufenamic acid were added to HCT116 AKR1C3 cells to determine the potency of these drugs at inhibiting the formation of 11β-PGF_2α_ from PGD_2_. SN33638 completely inhibited 11β-PGF_2α_ formation at an EC_50_ of 20.5 ± 5.3 nM (Figure 3D), while MPA was approximately 100-fold less potent (EC_50_ = 2.4 ± 1.6 μM) and indomethacin and flufenamic acid were inactive at all concentrations tested up to 30 μM. Addition of PGD_2_ or SN33638 to cells for 24 h had no effect on cell proliferation relative to unstimulated controls in all cell lines (data not shown).

### SN33638 inhibits testosterone production in high AKR1C3-expressing cell lines

**Figure 4:**
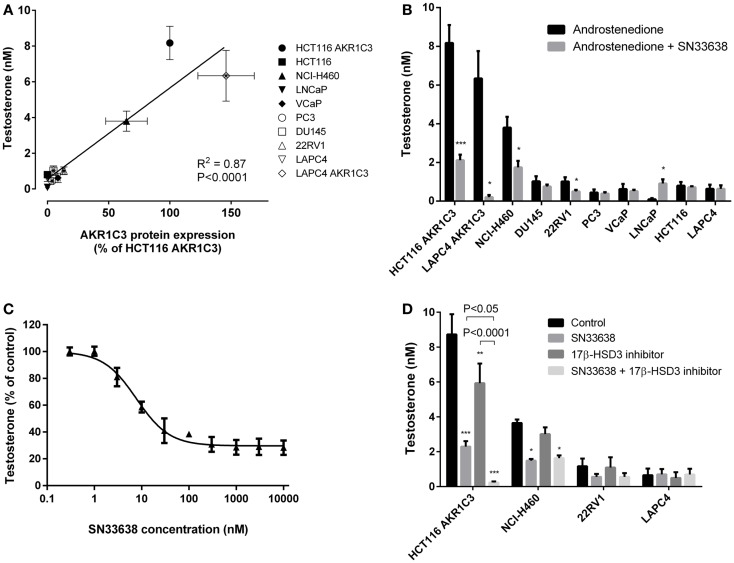
**Testosterone production correlates with AKR1C3 protein expression and can be partially inhibited by SN33638 in high AKR1C3-expressing cell lines**. Testosterone levels were determined by ELISA 24 h after stimulation with 28 nM androstenedione. **(A)** Correlation between testosterone levels (*n* = 3–6) and AKR1C3 protein expression (average of 2–3 independent lysates) in the prostate cancer, HCT116, and NCI-H460 cell lines. **(B)** Concentration of testosterone in the cell lines in the presence or absence of 10 μM SN33638 (*n* = 3–6). **(C)** EC_50_ plot of the inhibition of testosterone production by SN33638 in HCT116 AKR1C3 cells (*n* = 3). **(D)** Testosterone levels in HCT116 AKR1C3, NCI-H460, 22RV1, and LAPC4 cell lines following treatment with 10 μM SN33638 and 1 μM 17β-HSD3 inhibitor alone or in combination (*n* = 3–6). Bars or symbols represent the mean ± SEM. Statistical significance of correlation analysis was evaluated by Spearman’s rank-order correlation and of differences between mean values by Student’s *t*-test **(B)** or two-way ANOVA with Tukey’s multiple comparison analysis **(D)**.*P < 0.05; **P < 0.01; ***P < 0.001 vs. no inhibitor controls. Statistically significant differences between treatment groups are marked as indicated.

Since AKR1C3 has been implicated in the development of CRPC because of its ability to produce testosterone intratumorally from circulating androstenedione, we determined testosterone concentrations in the prostate cancer cell lines and known high and low AKR1C3-expressing cells after stimulation with androstenedione for 24 h. Similarly to 11β-PGF_2α_, testosterone levels positively correlated with AKR1C3 protein expression (*R* = 0.792, *P* < 0.01; Spearman’s rank-order correlation; Figure 4A). However, other than in LAPC4 AKR1C3-overexpressing cells, testosterone levels in the prostate cancer cell lines were no higher than in the negative control HCT116 (Figure 4B). Addition of 10 μM SN33638 significantly reduced testosterone levels in the high AKR1C3-expressing HCT116 AKR1C3 (74.2% inhibition, *P* < 0.001; Student’s *t*-test), LAPC4 AKR1C3 (96.8% inhibition, *P* < 0.05), and NCI-H460 cell lines (54.1% inhibition, *P* < 0.05), but only in one moderate AKR1C3-expressing cell line: 22RV1 (50.8% inhibition, *P* < 0.05). In LNCaP cells, testosterone levels were lower than the negative control after androstenedione stimulation alone, but were significantly increased on addition of 10 μM SN33638 to levels similar to those seen in the negative control and other prostate cancer cell lines (*P* < 0.05).

SN33638 potently inhibited testosterone production in HCT116 AKR1C3 cells with an EC_50_ of 7.4 ± 1.2 nM (Figure 4C) compared to MPA (EC_50_ = 4.3 ± 2.6 μM), indomethacin (EC_50_ > 30 μM), and flufenamic acid (EC_50_ > 30 μM); however, only approximately 70% of the testosterone produced in these cells could be inhibited by the AKR1C3 inhibitors. When testosterone formation was limited to that which was inhibitable by SN33638, the correlation with AKR1C3 protein expression (*R* = 0.951, *P* < 0.0001; Spearman’s rank-order correlation) was significantly improved (*z* = 2.561, *P* < 0.05; Steiger’s *z*-test) relative to the correlation between total testosterone and AKR1C3 protein expression. Since 17β-HSD3 can also convert androstenedione to testosterone, cells were treated with a non-cytotoxic dose (1 μM) of a 17β-HSD3 inhibitor (5-(3-bromo-4-hydroxybenzylidene)-3-(4-methoxyphenyl)-2-thioxothiazolidin-4-1) alone and in combination with SN33638. The 17β-HSD3 inhibitor significantly reduced testosterone levels in HCT116 AKR1C3 cells by 32.1% as a single agent (*P* < 0.01; two-way ANOVA with Tukey’s multiple comparison analysis) and by 97.3% in combination with SN33638 (*P* < 0.0001), an effect significantly greater than that achieved by SN33638 (73.7% inhibition, *P* < 0.05 vs. combination) or 17β-HSD3 inhibitor (*P* < 0.0001 vs. combination) treatment alone (Figure 4D). Inhibition of 17β-HSD3 had no effect on testosterone production either alone or in combination with SN33638 in the other cell lines tested (NCI-H460, 22RV1, and LAPC4).

### SN33638 does not substantially inhibit 17β-estradiol production in ER-positive breast cancer cell lines

**Figure 5:**
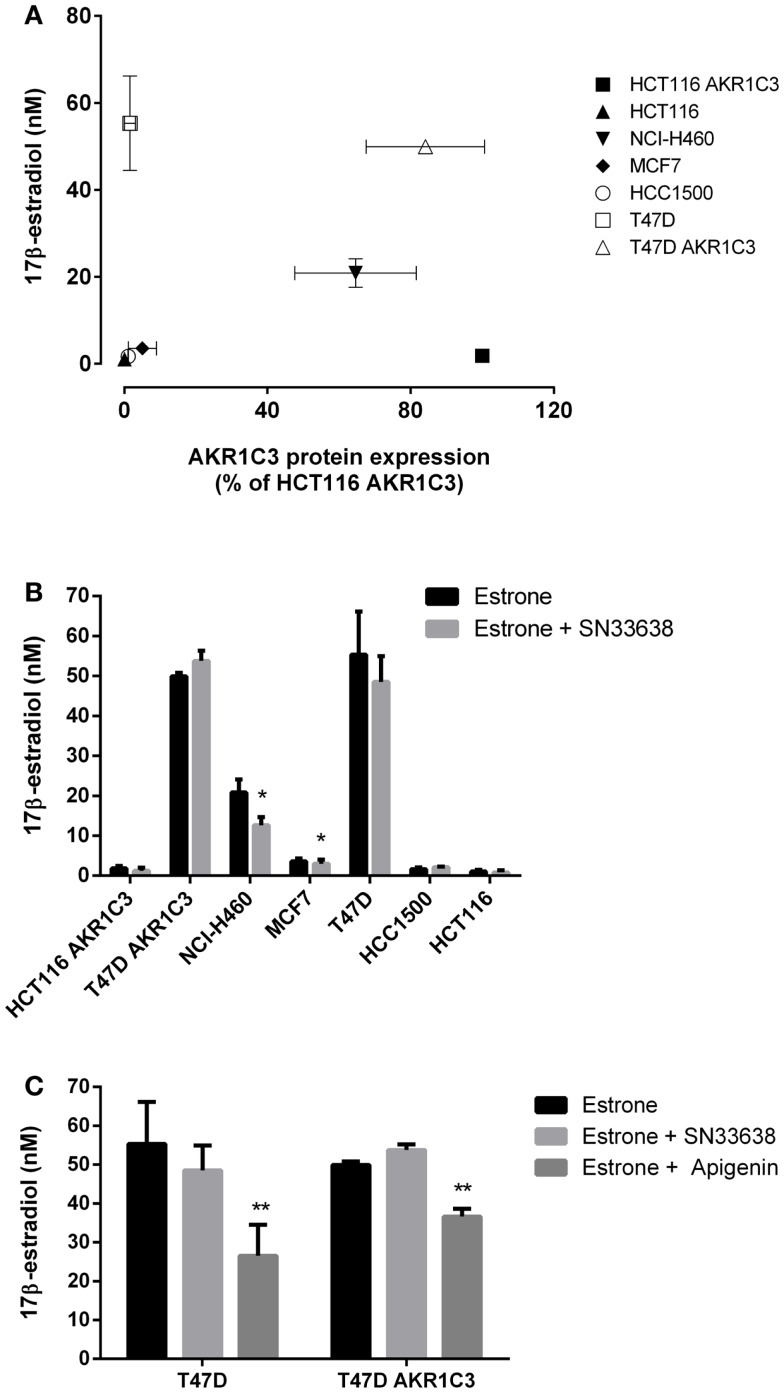
**SN33638 has little effect on estrone reduction to 17β-estradiol in ER-positive breast cancer cell lines**. 17β-estradiol levels were determined by ELISA 24 h after stimulation with 28 nM estrone. **(A)** Correlation between 17β-estradiol levels (*n* = 3–4) and AKR1C3 protein expression (average of 2–3 independent lysates) in ER-positive breast cancer, HCT116, and NCI-H460 cell lines. **(B)** Concentration of 17β-estradiol in the cell lines in the presence and absence of 10 μM SN33638 (*n* = 3–4). **(C)** 17β-estradiol levels in T47D and T47D AKR1C3 cells following treatment with 10 μM SN33638 and 30 μM apigenin alone or in combination (*n* = 3). Bars or symbols represent the mean ± SEM. Statistical significance of correlation analysis was evaluated by Spearman’s rank-order correlation and of differences between mean values by Student’s *t*-test **(B)** or one-way ANOVA with Dunnett’s multiple comparison analysis **(C)**. *P < 0.05; **P < 0.01 vs. no inhibitor controls.

17β-estradiol is predominantly produced from estrone by 17β-HSD1 in breast tissue in post-menopausal women (44). Although less catalytically efficient than 17β-HSD1, AKR1C3 can also convert estrone to 17β-estradiol (10, 12, 14), and therefore, may play a role in ER-positive breast cancer development. To investigate the activity of AKR1C3 in 17β-estradiol production, ER-positive breast cancer cells and known high and low AKR1C3-expressing cells were stimulated with estrone for 24 h prior to determination of 17β-estradiol levels by ELISA. Unlike 11β-PGF_2α_ and testosterone, 17β-estradiol levels did not significantly correlate with AKR1C3 protein expression in the cell lines investigated (*R* = 0.429; Spearman’s rank-order correlation; Figure 5A), indicating that AKR1C3 is not responsible for estrone reduction in some/all of these cell lines. 17β-estradiol levels were highest in T47D and T47D AKR1C3 cell lines, despite the large difference in AKR1C3 expression in this isogenic cell line pair. Addition of 10 μM SN33638 had no effect on the high 17β-estradiol levels in T47D and T47D AKR1C3 cells, but did inhibit 17β-estradiol formation by 16.0% in MCF7 cells (*P* < 0.05; Student’s *t*-test) and by 39.6% in NCI-H460 cells (*P* < 0.05) (Figure 5B). To determine if 17β-HSD1 was predominantly responsible for estrone reduction, the ER-positive breast cancer cell lines were also treated with apigenin, an inhibitor of 17β-HSD1 activity (45). Apigenin significantly inhibited 17β-estradiol formation in both T47D and T47D AKR1C3 cells (*P* < 0.05; one-way ANOVA with Dunnett’s multiple comparison analysis; Figure 5C), but had no inhibitory activity on MCF7 or HCC1500 cells (data not shown).

### SN33638 suppresses PSA levels in AKR1C3-expressing CRPC cells

**Figure 6:**
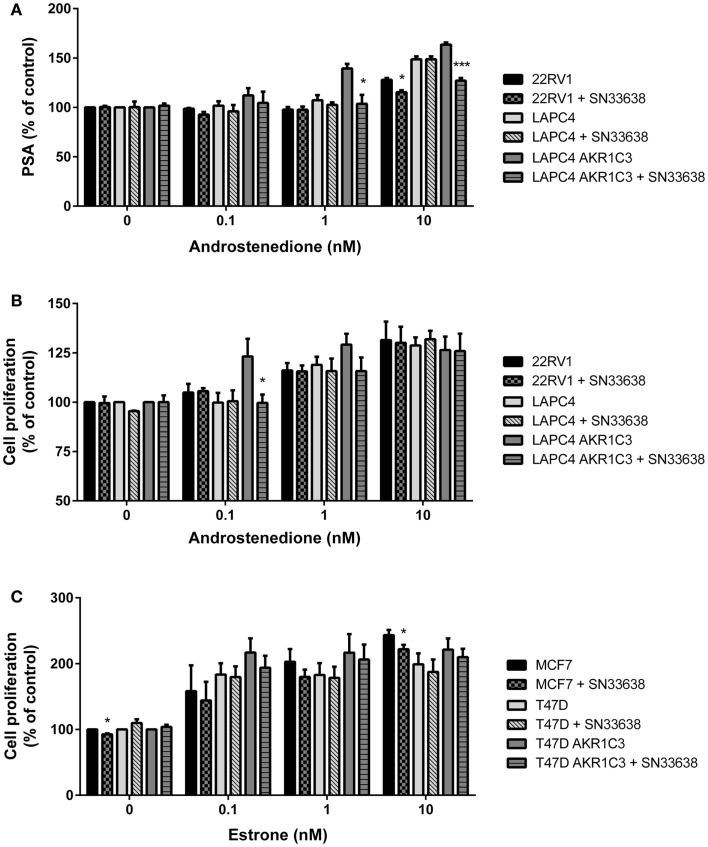
**SN33638 partially inhibits PSA expression and cell proliferation in LAPC4 AKR1C3 cells, but has limited activity on low-moderate AKR1C3-expressing CRPC cells and ER-positive breast cancer cells**. **(A)** PSA levels in CRPC cell lines 24 h after stimulation with androstenedione in the presence or absence of 10 μM SN33638 (*n* = 3). Cell proliferation in **(B)** CRPC cell lines and **(C)** breast cancer cell lines 5 days after stimulation with androstenedione or estrone and treatment with or without 10 μM SN33638 (*n* = 3–4). Bars represent the mean ± SEM. Significance of differences was evaluated by Student’s *t*-test. *P < 0.05; ***P < 0.001 vs. no inhibitor controls.

Next, PSA expression was determined in the CRPC cell lines that were sensitive to SN33638-mediated inhibition of testosterone (LAPC4 AKR1C3 and 22RV1 with LAPC4 wild-type cells acting as a negative control) as a biomarker of AR activity. Cells were stimulated with physiological concentrations of up to 10 nM androstenedione, with or without SN33638, for 24 h before PSA concentrations were determined. Androstenedione stimulation promoted PSA production in all three cell lines, with the highest PSA levels observed in LAPC4 AKR1C3 cells. SN33638 treatment significantly inhibited the formation of PSA in LAPC4 AKR1C3 cells that was attributable to stimulation with 1 nM (90.8% inhibition, *P* < 0.001; Student’s *t*-test) and 10 nM androstenedione (57.4% inhibition, *P* < 0.05), and in 22RV1 cells with 10 nM androstenedione (44.9% inhibition, *P* < 0.05), but had no significant effect on LAPC4 wild-type cells (Figure 6A).

### SN33638 has limited inhibitory activity on androstenedione or estrone-stimulated cell proliferation

Finally, we investigated whether inhibiting AKR1C3-dependent testosterone or 17β-estradiol production could prevent androstenedione- or estrone-stimulated cell proliferation in CRPC or ER-positive breast cancer cells. The AKR1C3-overexpressing and wild-type cell line pairs, 22RV1 and MCF7 cells were stimulated with physiological concentrations of androstenedione or estrone, with or without 10 μM SN33638, for 5 days. To limit the effect on cell proliferation of the testosterone or 17β-estradiol that could not be inhibited by SN33638 and due to the longer duration of the assay, lower concentrations of androstenedione, and estrone (up to 10 nM) were used than in the ELISA assays. SN33638 had no effect on unstimulated cell proliferation in all cell lines, except for a 7.3% reduction in MCF7 cells (*P* < 0.05; Student’s *t*-test), indicating it was largely non-cytotoxic at 10 μM. Stimulation of proliferation was observed with all concentrations of androstenedione in high AKR1C3-expressing LAPC4 AKR1C3 cells, but only at 1 and 10 nM in LAPC4 wild-type and 22RV1 cells. SN33638 significantly inhibited androstenedione-stimulated cell proliferation at 0.1 nM (101.4% inhibition, *P* < 0.05; Student’s *t*-test) and caused a substantial (45.9%) but non-significant reduction in stimulated cell proliferation with 1 nM androstenedione in LAPC4 AKR1C3 cells, but had no effect on androstenedione-stimulated cell proliferation in LAPC4 wild-type cells and 22RV1 cells (Figure 6B). Consistent with the lack of effect on 22RV1 cell proliferation, SN33638 had no inhibitory effect on 22RV1 testosterone levels 5 days after stimulation with up to 10 nM androstenedione (data not shown).

Estrone effectively stimulated cell proliferation in MCF7, T47D, and T47D AKR1C3 ER-positive breast cancer cells; however, this stimulation was not significantly inhibited by SN33638, other than a minor reduction in MCF7 cells at 10 nM estrone (14.9% inhibition, *P* < 0.05; Student’s *t*-test) (Figure 6C). Neither androstenedione nor estrone-stimulated cell proliferation in the HCT116 AKR1C3 and wild-type cell lines (data not shown).

## Discussion

AKR1C3 has recently been identified as a potential therapeutic target in both CRPC and ER-positive breast cancer, since it can promote intratumoral steroidogenesis to provide the hormones required for nuclear receptor activation and tumor progression (4, 6, 8–11). Several AKR1C3 inhibitors are currently being investigated [reviewed in Ref. (46)], including the oxopyrrolidinylphenylpiperidinosulfonamide compound SN33638 (Figure 1) (35). Here, we characterized the *in vitro* biological activity of SN33638 using a panel of CRPC and ER-positive breast cancer cell lines to determine if inhibition of AKR1C3 could prevent steroid hormone production, PSA expression, and cell proliferation.

We conducted a meta-analysis of *AKR1C3* mRNA expression data from multiple patient datasets that revealed that *AKR1C3* is upregulated in CRPC but downregulated in ER-positive breast cancer relative to normal prostate or breast tissues. The CRPC data confirmed previous reports (4, 8, 9, 47, 48), but our larger dataset showed *AKR1C3* upregulation was restricted to the upper quartile of CRPC samples. The lack of correlation between *AKR1C3* and *NQO1* expression in CRPC patient samples suggests that *AKR1C3* upregulation is not driven by an NRF2-mediated response to oxidative or electrophilic stress (32), but likely by a feedback loop in which low levels of androgens in patients undergoing androgen deprivation therapy upregulate *AKR1C3* to mediate intratumoral androgen levels (8, 48, 49). Previous studies on *AKR1C3* mRNA expression in small groups of invasive ductal carcinoma patients have generated conflicting data on whether *AKR1C3* is up- or downregulated relative to normal breast tissue (17, 20, 21). However, our meta-analysis of a much larger cohort of patients showed AKR1C3 is downregulated in ER-positive breast cancer, suggesting that AKR1C3 is unlikely to be a suitable therapeutic target for this disease.

Reduction of PGD_2_ to 11β-PGF_2α_ correlated linearly with AKR1C3 protein expression in the cancer cell line panel, indicating that PGD_2_ reduction provides a measurable biomarker of AKR1C3 activity in cells. This was confirmed by the presence of minimal 11β-PGF_2α_ concentrations in all cell lines treated with SN33638. A large concentration of SN33638 (10 μM) was used throughout these studies to ensure near-complete inhibition of AKR1C3 activity was achieved (Figure 3D), while maintaining cell viability and selectivity over other AKR1C isoforms (<14% inhibition against each isolated enzyme at 10 μM, unpublished data). AKR1C3 activity was variable across the cell line panel, with genetic overexpression required to produce activity similar to or greater than that observed in the known high AKR1C3-expressing NCI-H460 NSCLC cell line. Although VCaP cells have been used elsewhere to investigate the activity of small molecule inhibitors of AKR1C3 or RNAi in CRPC (6, 50), they displayed low AKR1C3 expression and activity in our studies. We therefore engineered an *AKR1C3*-overexpressing LAPC4 cell line to model the CRPC patients that had high *AKR1C3* mRNA expression.

Similarly to 11β-PGF_2α_ formation, the production of testosterone from androstenedione also correlated linearly with AKR1C3 protein expression, with AKR1C3 overexpression substantially increasing testosterone production, confirming that AKR1C3 plays an important role in this process. However, unlike 11β-PGF_2α_, testosterone was present in every cell line, including those that do not express AKR1C3, even after treatment with SN33638 or MPA. By limiting testosterone production to that which was SN33638-inhibitable, the correlation with AKR1C3 expression was significantly improved, suggesting that AKR1C3 was not the sole enzyme responsible for testosterone production, but that it does generate the majority of testosterone in high AKR1C3-expressing cell lines, especially LAPC4 AKR1C3, where 96.8% of testosterone was AKR1C3-dependent. AKR1C3-independent testosterone levels could be partially attributed to redundancy in androstenedione reduction by 17β-HSD3 (51) as evidenced in HCT116 AKR1C3 cells, where single agent treatment with a 17β-HSD3 inhibitor partially prevented testosterone formation, while combination treatment with a 17β-HSD3 inhibitor and SN33638 almost completely inhibited testosterone production. However, 17β-HSD3 inhibition had no effect on testosterone production, with or without SN33638, in NCI-H460, 22RV1, and LAPC4 cells, suggesting the involvement of one or more other 17β-HSD isoforms in testosterone production in these cells. These may include 17β-HSD types 1 and 15, which have both been shown to have a low capacity to convert androstenedione to testosterone (52, 53). Interestingly, testosterone levels in LNCaP cells were unexpectedly increased by SN33638 to similar levels to those detected in AKR1C3-negative cell lines. It is unclear why AKR1C3 inhibition promoted testosterone production in LNCaP cells, although it may be due to feedback upregulation of other 17β-HSD enzymes, which were likely to be present in LNCaP cells at lower levels than other CRPC cell lines given the low basal testosterone levels observed.

SN33638-mediated inhibition of testosterone production led to partial suppression of AR signaling as evidenced by reductions in PSA expression in LAPC4 AKR1C3 and 22RV1 cells and proliferation in LAPC4 AKR1C3 cells. Higher androstenedione concentrations were required to stimulate PSA (24 h incubation) than cell proliferation (5-day incubation) as more testosterone was produced with longer incubation times of androstenedione (data not shown). AKR1C3-independent testosterone concentrations also increased with longer incubation times and/or increased androstenedione concentrations, and are likely to account for the lack of effect of SN33638 on testosterone production or proliferation over the 5-day incubation in 22RV1 cells and on LAPC4 AKR1C3 cell proliferation with higher androstenedione concentrations. AKR1C3-independent testosterone production may also explain the observed stimulation of proliferation and PSA expression in the low AKR1C3-expressing LAPC4 cell line. These results suggest that AKR1C3 inhibition may prevent androstenedione-stimulated PSA expression or cell proliferation in CRPC cells, as previously shown for other small molecule inhibitors of AKR1C3 or RNAi (6, 47, 50), but because of the role of AKR1C3-independent testosterone (and DHT) in AR activation, the inhibitory effect is likely to be limited to CRPC cells that express high levels of AKR1C3 and to conditions where the cells are dependent on AKR1C3 for the generation of testosterone.

The greatest inhibitory effects of SN33638 were observed in the LAPC4 AKR1C3 cell line, which is an artificial experimental model. Whether similar effects of SN33638 would be observed in high AKR1C3-expressing wild-type cell lines is unknown, because of the dearth of CRPC cell lines available that express high levels of AKR1C3. This is surprising given the evidence here and elsewhere that AKR1C3 is upregulated in a subset of CRPC tumors (4, 8, 9, 47, 48). To address this issue, future studies of AKR1C3 inhibitors would benefit from utilizing CRPC patient-derived xenografts (54) from tumors that express high levels of AKR1C3. Such models could identify whether AKR1C3 inhibitors prevent the growth of tumors that originated from those patients that would be the most obvious candidates for treatment with this class of agents, but they would provide limited toxicology information since there is no AKR1C3 homolog in mice (55).

AKR1C3 has also been implicated in the development of ER-positive breast cancer because of its ability to convert estrone to 17β-estradiol (10, 11). However, we observed a poor correlation between AKR1C3 protein expression and estrone reduction to 17β-estradiol, while SN33638 was largely ineffective at preventing estrone reduction or estrone-stimulated cell proliferation in ER-positive breast cancer cell lines. T47D cells produced high levels of 17β-estradiol that could not be reduced by SN33638 treatment nor increased by AKR1C3 overexpression, but could be partially inhibited by apigenin or, as described previously, by the potent 17β-HSD1 inhibitor PBRM (56). This suggests a role for 17β-HSD1 in 17β-estradiol production in this cell line, with 17β-HSD7 and to a lesser extent 17β-HSD12 also likely to contribute since high levels of these enzymes are present in T47D cells (44, 57). The only breast cancer cell line in which SN33638 showed some inhibitory activity was MCF7, where we observed a 16% reduction in estradiol formation, similar to that previously reported for a steroidal lactone AKR1C3 inhibitor in this cell line (57). Despite MCF7 cells having low AKR1C3 protein expression, they appear to be partially dependent on AKR1C3 to produce 17β-estradiol, since the mRNA expression of *AKR1C3* in this cell line has been reported to be greater than that of the other 17β-HSD isoforms (types 1, 7, 12) that are capable of converting estrone to 17β-estradiol (57). Minor inhibition of estrone-stimulated cell proliferation was observed with SN33638 in MCF7 cells, but since inhibition was also observed in the absence of estrone stimulation, this effect may have been independent of the inhibition of estrone reduction. Overall, these results suggest that inhibition of AKR1C3 by SN33638 is ineffective at preventing ER-positive breast cancer growth.

In conclusion, our data suggests that while inhibition of AKR1C3 by SN33638 can prevent the conversion of PGD_2_ to 11β-PGF_2α_, its activity at preventing steroid hormone reduction and resultant CRPC and ER-positive breast cancer growth is limited due to the involvement of additional enzymes in testosterone and 17β-estradiol synthesis. AKR1C3 inhibitor therapy is therefore unlikely to be beneficial for the treatment of CRPC and ER-positive breast cancer, except possibly in the small subpopulation of CRPC patients with tumors that have upregulated *AKR1C3* expression and are dependent on AKR1C3 for producing the testosterone required for their growth.

## Author Contributions

Stephen M. F. Jamieson conceived and designed the experiments. Yarong Diana Yin and Melissa Fu performed the experimental studies. The majority of experiments in prostate cancer cell lines were conducted by Yarong Diana Yin and in breast cancer cell lines by Melissa Fu. Data was analyzed by Yarong Diana Yin, Melissa Fu, and Stephen M. F. Jamieson. Daniel M. Heinrich, Darby G. Brooke, and William A. Denny were responsible for the design of SN33638 and Daniel M. Heinrich and Darby G. Brooke for its synthesis. Stephen M. F. Jamieson drafted the figures, table, and manuscript. Yarong Diana Yin, Melissa Fu, Daniel M. Heinrich, Darby G. Brooke, and William A. Denny critically revised the paper and all authors approved the final version.

## Conflict of Interest Statement

The authors declare that the research was conducted in the absence of any commercial or financial relationships that could be construed as a potential conflict of interest.
